# Supplementary material for: ANGPTL7, a therapeutic target for increased intraocular pressure and glaucoma
Source: Commun Biol. 2022 Oct 3;5:1051. doi: 10.1038/s42003-022-03932-6 (PMC9529959; doi:10.1038/s42003-022-03932-6)
Supplement: Supplementary file 2 — Supplementary Information [file 42003_2022_3932_MOESM2_ESM.pdf]

SUPPLEMENTARY METHODS:

Glaucoma phenotype definition

GHS, SINAI: ICD-based glaucoma case definition in GHS and SINAI required an in-patient diagnosis or ≥ 2 outpatient diagnoses of ICD-10-H40 in the EHR. Individuals with only 1 outpatient diagnosis were excluded from the analysis. ICD-based controls for glaucoma were defined as individuals who were not cases or excluded *(50, 51)*.

UKB: Glaucoma ICD-based definitions of cases in UKB required one or more of the following: a) ≥ 1 diagnosis in inpatient Health Episode Statistics (HES) records, b) a cause-of-death diagnosis in death registry, c) ≥ 2 diagnoses in outpatient data (READ codes mapped to ICD10). Since we had self-reported diagnoses available for glaucoma in UKB, we combined ICD-based and self-reported glaucoma to define cases. Individuals were considered cases if they: identified 'glaucoma' from the eye problems or disorders list in the touchscreen questionnaire (UKB field ID: 6148) or, stated they had glaucoma in the verbal interview (UKB field ID: 20002) or were a case for ICD-10 H40 glaucoma. Normal controls for glaucoma in UKB were defined as individuals who did not report having glaucoma in the touchscreen (UKB field ID: 6148) or the verbal interview (UKB field ID: 20002), and were defined as controls for ICD-based glaucoma as described above.

POAAGG: A detailed description of criteria used to define glaucoma cases in POAAGG is provided elsewhere *(49)*. In brief, POAG cases were defined as having an open iridocorneal angle and characteristic glaucomatous optic nerve findings in one or both eyes, characteristic visual field defects and all secondary causes of glaucoma excluded. Controls in POAAGG were defined as subjects older than 35, without high myopia (greater than -8.00 diopters) or presbyopia (+8.00

diopters), a family history of POAG, abnormal visual field, IOP greater than 21 mmHg, neuroretinal rim thinning, excavation, notching or nerve fiber layer defects, optic nerves asymmetry or a cup to disc ratio between eyes greater than 0.2. Additional controls for POAAGG were identified from the Penn Medicine Biobank as individuals without ICD-9 diagnoses for glaucoma.

MALMO: Glaucoma cases were defined as individuals with ≥ 1 in-patient and ≥ 2 outpatient diagnoses for ICD8: 375, ICD9: 365 or ICD10: H40. Individuals with only 1 outpatient diagnosis were excluded from the analysis. Controls were individuals who were not cases or excluded.

EstBB: Glaucoma cases were defined as individuals with at least 2 records of ICD-10 H40 (and its descendants) and controls were individuals that without a diagnosis for ICD-10 H40-H42, ICD-10 H44.5 and ICD-10 Q15.0. Individuals with only 1 ICD-10 H40 code were excluded from the analysis.

HUNT: Glaucoma cases were defined as individuals with an ICD-10 H40 or an ICD-9 365 diagnosis code at 2 separate outpatient encounters or 1 in-patient encounter. Individuals with only 1 outpatient encounter were excluded from the analysis. Individuals were excluded from controls if they had any of the following codes: ICD-10 H40-H42, ICD-10 H44.51, ICD-10 Q15.0 / ICD-9 365, ICD-9 377.14 or ICD-9 360.42.

CGPS-CCHS: Diagnoses of glaucoma were collected from the national Danish Patient Registry from January 1st, 1977 to March 1st, 2018. The National Danish Patient Registry has information

on all patient contacts with all clinical hospital departments in Denmark, including emergency wards and outpatient clinics (from 1994). Glaucoma cases were defined as individuals with ICD-10 H40 and/or ICD8 375, and controls were participants without any of these codes.

FinnGen: The FinnGen analysis used was finngen_r3_H7_GLAUCOMA. Glaucoma cases were defined as individuals with ICD-10 codes of H40-H42 in the electronic health records and controls were individuals without any of these codes.

Exome sequencing

High coverage whole-exome sequencing was performed at the Regeneron Genetics Center *(50, 51)*. NimbleGen probes (VCRome) or a modified version of the xGen design available from Integrated DNA Technologies (IDT) were used for target sequence capture. Sequencing was performed using 75-bp paired-end reads on Illumina v4 HiSeq 2500 or NovaSeq instruments. Sequencing had a coverage depth (ie, number of sequence-reads covering each nucleotide in the target areas of the genome) sufficient to provide greater than 20x coverage over 85% of targeted bases in 96% of VCRome samples and 20x coverage over 90% of targeted bases in 99% of IDT samples. Sequence read alignment and variant calling was based on the GRCh38 Human Genome reference sequence. Ensembl v85 gene definitions were used to determine the functional impact of single nucleotide variants and insertion-deletions. Predicted LOF genetic variants included (a) insertions or deletions resulting in a frameshift, (b) insertions, deletions or single nucleotide variants resulting in the introduction of a premature stop codon or in the loss of the transcription start site or stop site, and (c) variants in donor or acceptor splice sites. Missense variants were classified for likely functional impact according to the number of *in silico* prediction

algorithms that predicted deleteriousness using SIFT *(58)*, Polyphen2_HDIV *(59)* and Polyphen2_HVAR *(59)*, LRT *(60)* and MutationTaster *(61)*. We aggregated rare variants for gene burden testing as previously described *(62)*. Briefly, rare variants were collapsed by gene region, such that individuals who are homozygous reference for all variants are considered homozygous reference, heterozygous carriers of any aggregated variant are considered heterozygous, and only minor allele homozygotes for an aggregated variant are considered as minor allele homozygotes. Genotypes were not phased to consider compound heterozygotes in burden testing. For each gene, we considered two categories of masks: a strict burden of rare pLOFs and a more permissive burden of rare pLOFs and likely deleterious missense variants. For each of these groups, we considered five separate burden masks per gene, based on the frequency of the alternative allele of the variants that were screened in that group: MAF ≤ 1%, MAF ≤ 0.1%, MAF ≤ 0.01%, MAF ≤ 0.001%, and singletons only. For the purposes of gene burden testing, the singleton mask includes minor allele homozygotes if no other variant carriers are observed in the dataset.

Genotyping

UKB: DNA samples were genotyped as described previously *(63)* using the Applied Biosystems UK BiLEVE Axiom Array (N=49,950) or the closely related Applied Biosystems UK Biobank Axiom Array (N=438,427). Genotype data for variants not included in the arrays were inferred using three reference panels (Haplotype Reference Consortium, UK10K and 1000 Genomes Project phase 3) as described previously *(63)*.

GHS, SINAI, MALMO and POAAGG: For SINAI, MALMO and POAAGG, DNA from participants was genotyped on the Global Screening Array (GSA) and for GHS, genotyping was

done on either the Illumina OmniExpress Exome (OMNI) or GSA. MALMO and SINAI were imputed to the HRC reference panel using the University of Michigan Imputation Server. POAAGG was imputed to the 1KG reference panel. GHS was imputed to the TOPMed reference panel (stratified by array) using the TOPMed Imputation Server. Prior to imputation, we retained variants that had a MAF ≥ 0.1%, missingness < 1% and HWE $P > 10^{-15}$. Following imputation for GHS, data from the OMNI and GSA datasets were merged for subsequent association analyses, which included an OMNI/GSA batch covariate, in addition to other covariates described below.

HUNT: The Trøndelag Health Study (HUNT) consists of four different population-based health surveys conducted in the county of Nord-Trøndelag, Norway over approximately 35 years (HUNT1 [1984-1986], HUNT2 [1995-1997], HUNT3 [2006-2008]), HUNT4 [2017-2019] *(45)*. At each survey, the entire adult population (≥ 20 years) was invited to participate by completing questionnaires, attending clinical examinations and interviews. Participation rates in HUNT1, HUNT2, HUNT3 and HUNT4 were 89.4%, 69.5% and 54.1%, 54.0%, respectively. Taken together, the study included more than 120,000 different individuals from Nord-Trøndelag County.

DNA from 71,860 HUNT samples was genotyped using Illumina HumanCoreExome arrays (HumanCoreExome12 v1.0, HumanCoreExome12 v1.1 and UM HUNT Biobank v1.0). Genotyping and quality control have been previously described *(64)*. Imputation was performed on samples of recent European ancestry using Minimac3 (v2.0.1, http://genome.sph.umich.edu/wiki/Minimac3) *(65)* and a merged reference panel that was constructed by combining the Haplotype Reference Consortium panel (release version 1.1) *(66)* and a local reference panel based on 2,202 whole-genome sequenced HUNT study participants.

EstBB: Genotyping of DNA samples from the Estonian Biobank was done at the Core Genotyping Lab of the Institute of Genomics, University of Tartu using the Illumina Global Screening Arrays (GSAv1.0, GSAv2.0, and GSAv2.0_EST). At the time of this study altogether 155,772 samples were genotyped and then PLINK format files were created using Illumina GenomeStudio v2.0.4. During the quality control all individuals with call-rate < 95% or mismatching sex that was defined based on the heterozygosity of X chromosome and sex in the phenotype data, were excluded from the analysis. Variants were filtered by call-rate < 95% and HWE p-value $< 1 \times 10^{-4}$ (autosomal variants only). Variant positions were updated to Genome Reference Consortium Human Build 37 and all variants were changed to be from TOP strand using reference information provided by Dr. Will Rayner from the University of Oxford (https://www.well.ox.ac.uk/~wrayner/strand/). After QC the dataset contained 154,201 samples. Before imputation variants with MAF<1% and Indels were removed. Prephasing was done using the Eagle v2.3 software *(67)* (number of conditioning haplotypes Eagle2 uses when phasing each sample was set to: --Kpbwt=20000) and imputation was carried out using Beagle v.28Sep18.793 *(68, 69)* with an effective population size ne = 20,000. As a reference, Estonian population specific imputation reference of 2,297 whole genome sequenced (WGS) samples was used *(70)*.

CGPS-CCHS: *ANGPTL7* Gln175His was genotyped with the Illumina HumanExome BeadChip (n=19,719), and Arg177* was genotyped by Taqman (n=113,604). All Arg177* heterozygotes were verified by Sanger sequencing.

FinnGen: Individuals in FinnGen were genotyped with Illumina and Affymetrix chip arrays (Illumina Inc., San Diego, and Thermo Fisher Scientific, Santa Clara, CA, USA). Imputation was performed using the population specific SISu v3 imputation reference panel of 3,777 whole genomes. Additional details on genotyping and imputation can be found at: https://finngen.gitbook.io/documentation/.

Genetic association analyses in UKB, GHS, SINAI, MALMO and POAAGG

Association analyses in each study were performed using the genome-wide linear (for IOP) or Firth logistic (for glaucoma) regression test implemented in REGENIE *(52)*. We included in step 1 of REGENIE (i.e. prediction of individual trait values based on the genetic data) directly genotyped variants with a minor allele frequency (MAF) > 1%, < 10% missingness, Hardy-Weinberg equilibrium test $P > 10^{-15}$ and linkage-disequilibrium (LD) pruning (1000 variant windows, 100 variant sliding windows and $r^2 < 0.9$). The association model used in step 2 of REGENIE included as covariates (i) age, age^2, sex, age-by-sex and age^2-by-sex; (ii) 10 ancestry-informative principal components (PCs) derived from the analysis of a set of LD-pruned (50 variant windows, 5 variant sliding windows and $r^2 < 0.5$) common variants from the array (imputed for the GHS study) data generated separately for each ancestry; (iii) an indicator for exome sequencing batch (GHS: three batches; UKB: six IDT batches); and (iv) 20 PCs derived from the analysis of exome variants with a MAF < 1% also generated separately for each ancestry.

Within each study, association analyses were performed separately for individuals of African (AFR) and European (EUR) ancestry, when available. We determined continental ancestries by projecting each sample onto reference principal components calculated from the HapMap3

reference panel. Briefly, we merged our samples with HapMap3 samples and kept only SNPs in common between the two datasets. We further excluded SNPs with MAF < 10%, genotype missingness > 5% or Hardy-Weinberg Equilibrium test $P < 10^{-5}$. We calculated PCs for the HapMap3 samples and projected each of our samples onto those PCs. To assign a continental ancestry group to each non-HapMap3 sample, we trained a kernel density estimator (KDE) using the HapMap3 PCs and used the KDEs to calculate the likelihood of a given sample belonging to each of the five continental ancestry groups. When the likelihood for a given ancestry group was > 0.3, the sample was assigned to that ancestry group. When two ancestry groups had a likelihood > 0.3, we arbitrarily assigned AFR over EUR, Admixed American (AMR) over EUR, AMR over East Asian (EAS), South Asian (SAS) over EUR, and AMR over AFR. Samples were excluded from analysis if no ancestry likelihoods were > 0.3, or if more than three ancestry likelihoods were > 0.3. Results were subsequently meta-analyzed across studies and ancestries using an inverse variance-weighted fixed-effects meta-analysis.

Genetic association analyses in HUNT, EstBB, CGPS-CCHS and FinnGen

HUNT: Association analyses were conducted using SAIGE *(53)*. Models were adjusted for birth year, birth year squared, sex, birth year-by-sex interaction, genotyping batch and four principal components (PCs). PCs were computed using PLINK. Additionally, the analyses were restricted to participants of European ancestry.

EstBB: We conducted the GWASes using the SAIGE software *(53)* with mixed-model logistic regressions and adjusting the analyses for the first four principal components of the genotype matrix, as well as for age, age squared and sex.

CGPS-CCHS: The associations between *ANGPTL7* genotype and glaucoma were tested with logistic regression, adjusted for sex and age.

FinnGen: GWAS in FinnGen was conducted using the SAIGE software and adjusted for sex, age, first 10 principal components and genotyping batch.

Phenome-wide association analysis for *ANGPTL7* pLOF and missense variants

We undertook a phenome-wide analysis of the association of an aggregate of pLOF and missense variants in *ANGPTL7* with hundreds of continuous traits or disease outcomes in the GHS and UKB studies. Results were available for 24,082 outcomes across the two cohorts. To control for the number of statistical tests performed, associations were considered statistically significant if the association p-value met a Bonferroni correction for 24,082 tests, that is $P < 2 \times 10^{-6}$ (corresponding to a p-value threshold of 0.05 divided by 24,082 statistical tests).

Continuous traits and disease outcomes were defined as described below. In the UKB study, for continuous traits, the values of biomarker, imaging variables or other continuous traits measured during one of the UKB visits or their averages within a given study visit or across study visits were used as outcomes. For binary disease outcomes, case status definition required one or more of the following criteria to apply (a) self-reported disease status or use of medication at digital questionnaire or interview with a trained nurse or (b) EHR of inpatient encounters from the UK National Health Service Hospital Episode Statistics database coded using the ICD-10 coding system. For each binary outcome, controls were individuals without any of the criteria for case

definition. In the GHS study, for binary disease outcomes, case status definition required one or more of the following criteria to apply: (1) a problem-list entry of the ICD-10 diagnosis code, (2) an inpatient hospitalization-discharge ICD-10 diagnosis code, or (3) an encounter ICD-10 diagnosis code entered for 2 separate outpatient visits on separate calendar days. Controls were individuals without any of the criteria for case definition. Individuals were excluded if they had the relevant ICD-10 code associated with only one outpatient encounter. For continuous traits, data cleaning was performed by removing non-physiologic lab values, invalid or contaminated specimens, and those that were over 5 times the upper limit of normal. Then the minimum, median, and maximum laboratory result values over the duration of follow-up were derived for each patient and used as outcomes.

Small interfering RNA molecules

Small interfering RNAs molecules used in this study were synthesized by Alnylam Pharmaceuticals, Inc. (Cambridge, MA) as described by Nair et al *(71)*. The identities and purities of all oligonucleotides were confirmed by electrospray ionization mass spectroscopy and ion exchange high-performance liquid chromatography, respectively. These are siRNA molecules that contain modified bases (2' mods) and such molecules are conjugated to a proprietary ocular targeting agent/moiety. Concentration of siRNA molecules used in this study were 15mg/ml.

Derivation of mean corneal refractive power and astigmatism from refractometry traits

Corneal refractive power and corneal astigmatism were derived from the autorefractometry and keratometry data available in UKB as previously described *(72)*. Briefly, corneal astigmatism was defined corneal power along strong meridian minus corneal power along weak meridian at 3mm

diameter, whereas the corneal power was the average of these two values for each eye. Refractive astigmatism is defined as the mean cylindrical power between both eyes *(73)*.

Bulk RNAseq

For the eye atlas bulk RNA sequencing, KAPA Stranded mRNA-Seq Kit by Illumina (https://www.illumina.com/) was used for the library preparation. Pippin HT instrument was used to select 400-600bp fragments prior to sequencing on Illumina Hiseq 2500 using pair-end 2*100 base pair protocol. The sequence alignment was performed using ArrayStudio RNA-seq pipeline (https://www.qiagen.com/). More specifically, Human.B37.3 was used as genome reference and OmicsGene20130723 was used as gene model.

Generation of *Angptl7*$^{-/-}$ mice

The genetically engineered *Angptl7-/-* mouse strain was created using Regeneron's VelociGene technology *(74, 75)*. Briefly, mouse embryonic stem cells (50% C57BL/6NTac; 50% 129S6/SvEvTac; and Crb1$^{+/+}$) were targeted for ablation of a 571 base pair region of the *Angptl7* locus, beginning 153 base pairs upstream of the start ATG (mm10 chr4:148,499,872-148,500,442). A self-deleting Hygromycin selection cassette was targeted to the deletion for selection in embryonic stem cells. Heterozygous targeted cells were microinjected into 8-cell embryos from Charles River Laboratories Swiss Webster albino mice, yielding F0 VelociMice that were 100% derived from the targeted cells *(75)*. These mice were subsequently bred to homozygosity and maintained in the Regeneron animal facility during the study period. The resistance cassette was removed during F0 breeding using self-deleting technology.

Anterior segment imaging using optical coherence tomography

Mice were anesthetized with 0.1 mg/kg of a ketamine/xylazine mixture (12 mg/ml and 0.5 mg/ml, respectively) and one drop of topical proparacaine (0.05%, sterile) on the eyes. After a minute, proparacaine was wiped off of the eyes and images of anterior segment of mice were collected using the infrared (IR) and optical coherence tomography + IR (OCT+IR) options on the Heidelberg Spectralis machine. Following parameters were used to capture images: sensitivity (42), position (-0.00 mm), ART (6 frames), size of scan (large), width and height (15 degrees × 10 degrees), and number of sections (81) were the same for all OCT and OCT+IR images. In addition, we used the sclera option for capturing OCT+IR images of mouse eyes. After acquiring images, mice were put on a warming station and monitored until they were fully awake and exhibiting normal behavior. Corneal thickness was measured using the Heidelberg Eye Explorer (version 1.5.9.0) by three experts in mouse eye anatomy from OCT images of the center of the cornea (section 41/81 at zoom 800%). The measurements from all three individuals were collated in the final plot.

In situ hybridization using *RNAscope*

The expression pattern of TM single cell cluster specific gene expression in the human donor eye was determined by in situ hybridization using RNAscope® according to manufacturer's specifications (Advanced Cell Diagnostics). Briefly, 10% NBF fixed and paraffin embedded human donor eye cups were cut into 5 to 10μm sections and mounted on SUPERFROST® Plus glass slides. For RNAscope, slides were baked on slide warmer for 1 hour at 60ºC and deparaffinized for 20 minutes. Tissue sections then underwent 10 minutes of Pretreat 1- RNAscope hydrogen peroxide treatment (ACD, 320037) at room temperature, followed by 20 minutes of boiling at 90ºC in Pretreat 2- target retrieval treatment (ACD, 320043) in Oster Steamer (IHC

World, LLC, Model 5709) and 30 minutes of Pretreat 3- RNAscope protease plus treatment (ACD, 320037) at 40°C in a HybEZ Oven (ACD, 310010). Tissue sections were then incubated with DNaseI for 10 minutes at 40°C to reduce potential background from probes binding to genomic DNA. Tissue sections were then washed five times with water, hybridized with RNAscope probes for 2 hours at 40°C and the remainder of the manufacturer's assay protocol was implemented (ACD, 322360) from Amplified 1 to Amplified 6. The slides were washed twice (two minutes each at room temperature) with RNAscope wash buffer (ACD, 310091). Signal was detected by incubation with Red working solution (1:60 ratio of Red B to Red A) at room temperature for 10 minutes in the absence of light, followed by washing the slides in water several times and viewing under microscope. In some experiments, fluorescent signals were visualized and captured using an open-field Nikon Eclipse Ti-E microscope.

Supplementary Table 1: Number of samples across cohorts included in IOP and glaucoma analyses.

S1A:

IOP (EUR)

Cohort	Data Type	# Samples
UKB	Array	101,590
	Imputed	108,120
	Exome	101,678
GHS	Imputed	28,977
	Exome	27,529

S1B:

Glaucoma (EUR)

Cohort	Data Type	# Cases	# Controls
UKB	Array	11,494	373,246
	Imputed	12,377	400,978
	Exome	11,502	373,538
GHS	Imputed	8,032	114,171
	Exome	7,562	110,602
SINAI	Array	409	9,178
	Imputed	409	9,178
	Exome	409	9,178
MALMO	Array	2,395	26,062
	Imputed	2,395	26,062
	Exome	2,395	26,062
FinnGen(R3)	Array/Imputed	3,463	93,036
EstBB	Array/Imputed	7,629	128,075
HUNT	Array/Imputed	3,874	64,541
CGPS-CCHS	Taqman	1,863	111,741

S1C:

IOP (AFR)

Cohort	Data Type	# Samples
UKB	Array	4,132
	Imputed	4,405
	Exome	4,114
POAAGG	Array	3,282
	Imputed	3,282
	Exome	3,167

S1D:

Glaucoma (AFR)

Cohort	Data Type	# Cases	# Controls
UKB	Array	449	7,374
	Imputed	481	7,922
	Exome	448	7,328
SINAI	Array	1,261	10,270
	Imputed	1,261	10,270
	Exome	1,261	10,270
POAAGG	Array	3,590	4,184
	Imputed	3,590	4,184
	Exome	3,444	4,052

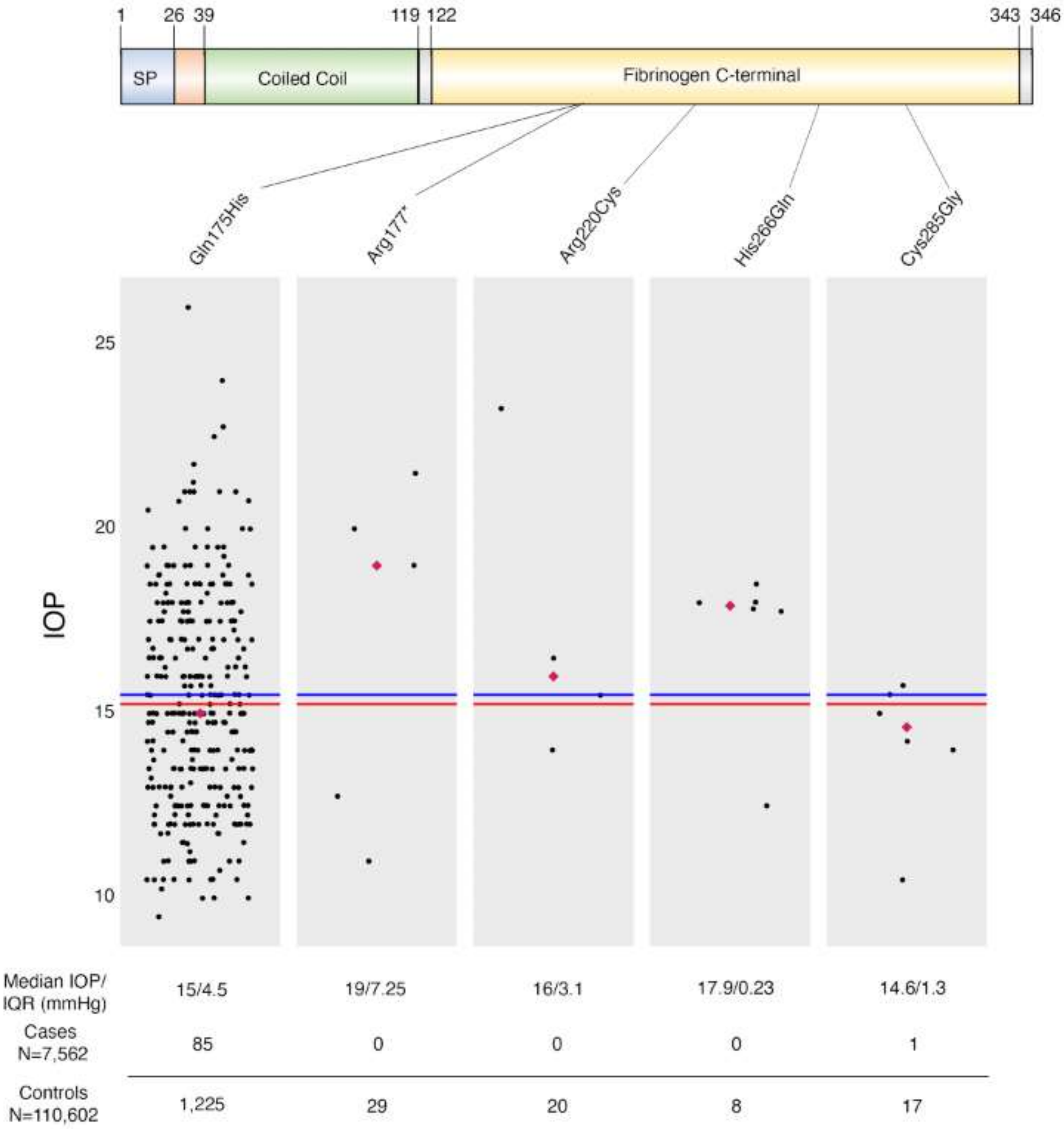

Supplementary Figure 1: Missense and predicted loss-of-function (pLOF) variants in *ANGPTL7* and IOP levels in individuals of European descent in GHS. The plots represent Goldmann-correlated IOP (mean of both eyes) levels in carriers of 1 pLOF and 4 missense variants in *ANGPTL7* that are predicted deleterious by five different algorithms and have at least five carriers amongst the 27,529 exome-sequenced individuals with IOP measurements in GHS. The

median IOP level across carriers of all 32 pLOF and predicted-deleterious missense *ANGPTL7* variants (15.25 mmHg) is indicated by the red line, and the median IOP in non-variant carriers (15.50 mmHg) is indicated by the blue line. Magenta diamonds mark the median IOP in carriers of each variant. Beneath the plots is the median and interquartile range of IOP and the numbers of variant carriers diagnosed with glaucoma or controls in GHS (n=118,164).

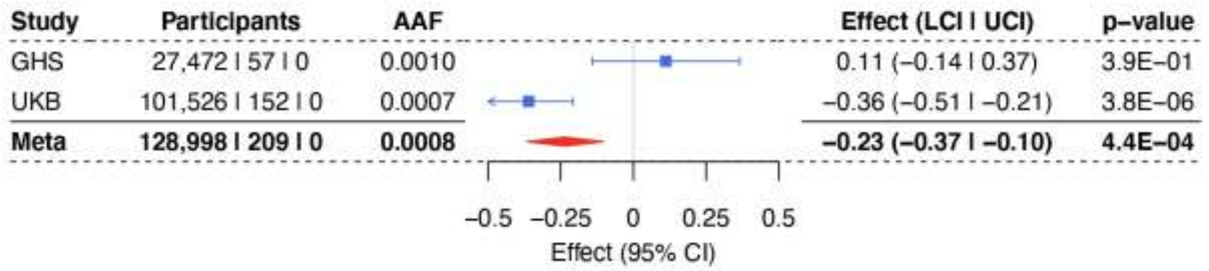

Supplementary Figure 2: Meta-analysis of *ANGPTL7* aggregate of predicted loss-of-function and deleterious missense variants (MAF < 1%), excluding Gln175His and Arg177*, with IOP. A total of 61 variants were present in the burden test.

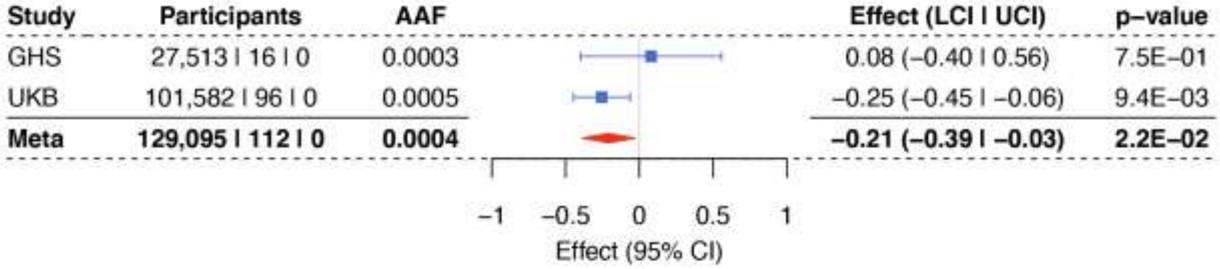

Supplementary Figure 3: Meta-analysis of *ANGPTL7* aggregate of predicted loss-of-function variants only (MAF < 1%) with IOP. Arg177* is included in this aggregate of 15 variants.

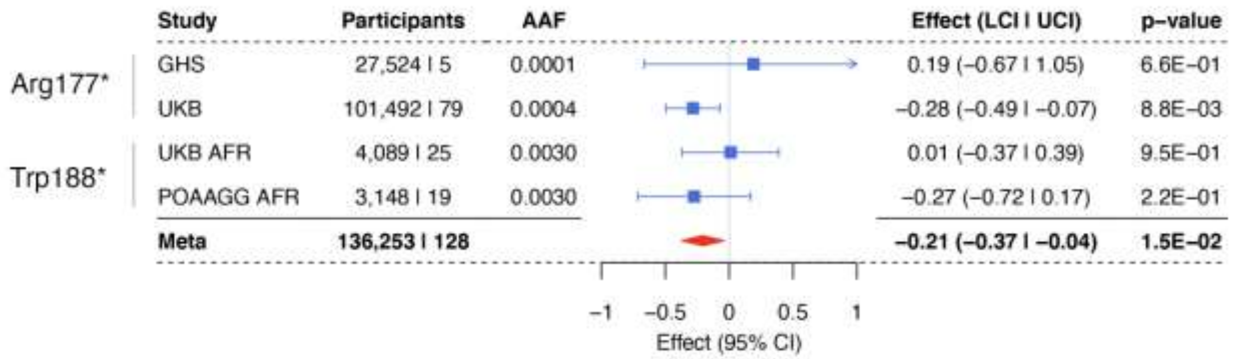

Supplementary Figure 4: Cross-ancestry meta-analysis of Arg177* and Trp188* with IOP.

AFR = African ancestry cohorts.

Supplementary Table 2: Association of *ANGPTL7* aggregate of pLOF and deleterious missense variants with ocular traits of interest in UKB.

Trait	P-value	Effect in SD (LCI | UCI)
CH (mean of both eyes)	7.50E-03	-0.06[-0.10 | -0.16
Moderate to low myopia (20262)	5.20E-02	1.1 [1.0 | 1.3]
ICD10 H52.1: Myopia	2.90E-01	1.2 [0.85 | 1.7]
High myopia (20262)	8.30E-01	0.97 [0.76 | 1.2]
Mean Spherical Equivalent (mean of both eyes)	1.40E-01	-0.032 [-0.074 | 0.010]
Corneal Astigmatism 6mm (left eye)	2.80E-01	-0.027 [-0.075 | 0.022]
Corneal Astigmatism 6mm (right eye)	5.80E-01	-0.014 [-0.062 | 0.035]
Corneal Astigmatism 3mm (left eye)	9.80E-01	0.00071 [-0.045 | 0.046]
Corneal Astigmatism 3mm (right eye)	1.00E+00	-0.00014 [-0.045 | 0.045]
Refractive Astigmatism	9.10E-01	0.0025 [-0.041 | 0.046]

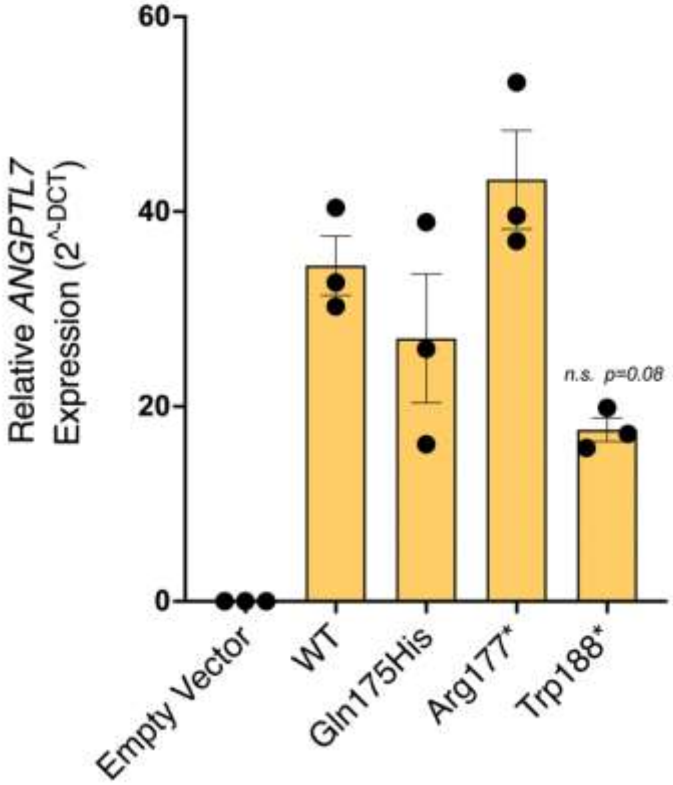

Supplementary Figure 5: Relative expression of WT and variant ANGPTL7 mRNA in a HEK293 cell line. qPCR shows mRNA levels of ANGPTL7 wild type, ANGPTL7 Gln175His, Arg177* and Trp188*, after transfection in HEK293. The experiment shows the average expression of ANGPTL7 and its variants in three biological replicates. Technical replicates (n=3) were run for all three qPCR replicates. P-values were calculated by one-way ANOVA with Tukey's post hoc analysis. Data are represented as mean and error bars reflect the standard error of the mean.

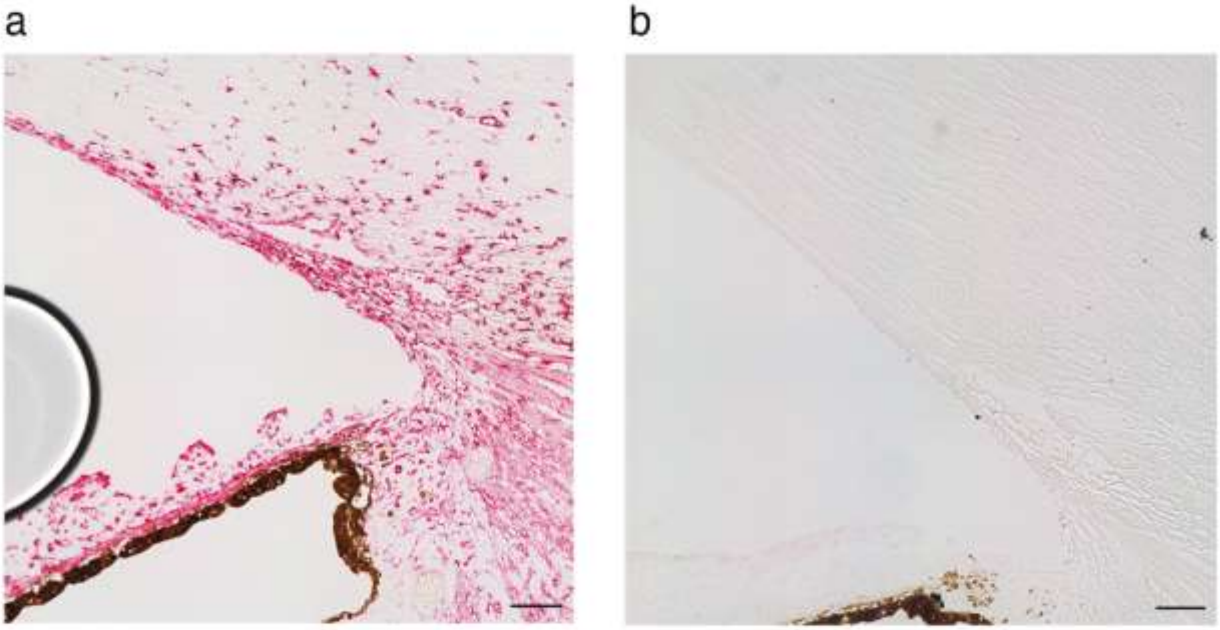

Supplementary Figure 6: Positive and negative controls for in situ hybridization of *ANGPTL7* in human eyes. Brightfield images showing following probes (a) Positive control- Ubc (red), (b) Negative control- DapB probe. Scale bars represent 100 μm.

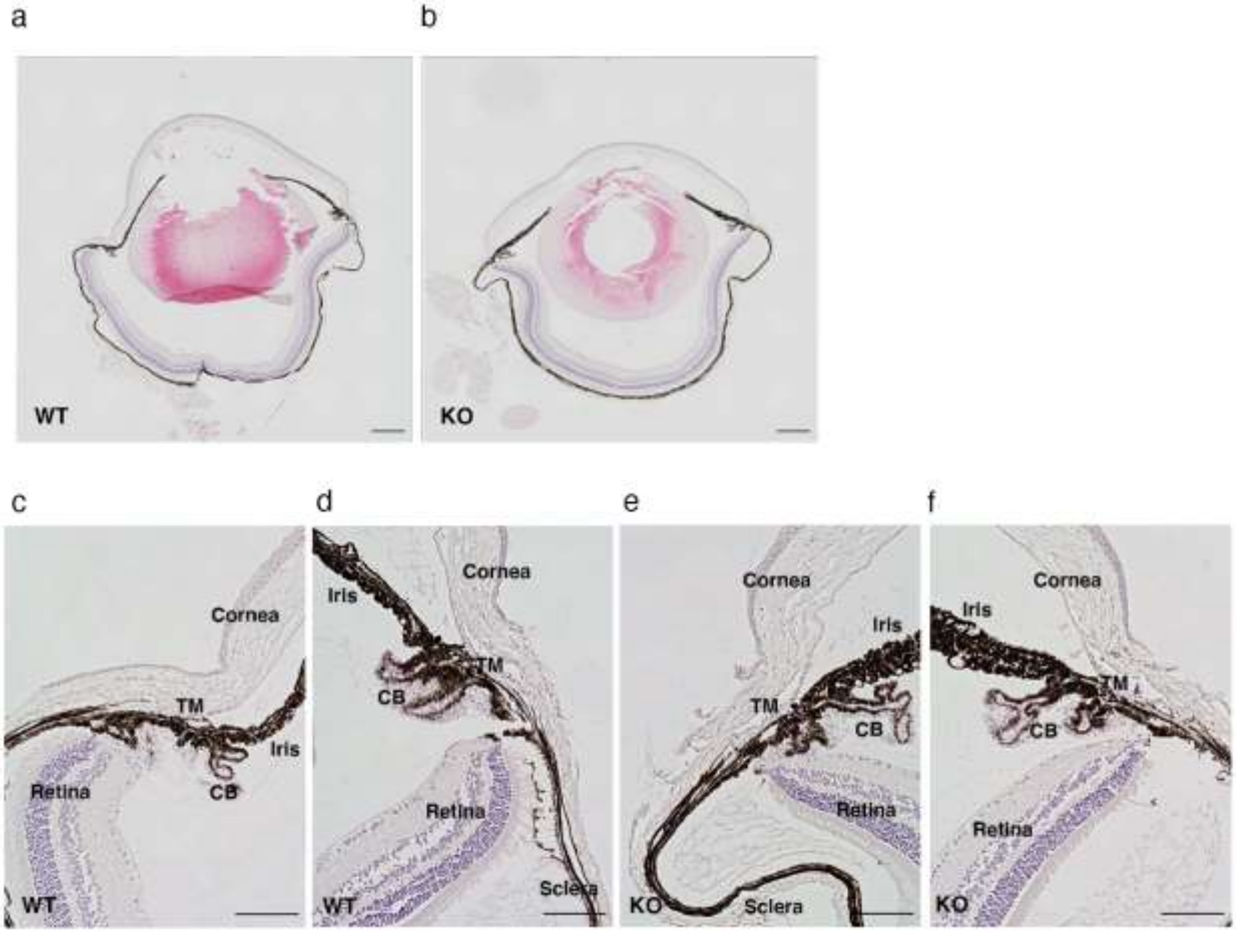

Supplementary Figure 7: Histological analysis of eyes from WT and *Angptl7* KO mice. (a, b) Hematoxylin and eosin (H&E) staining of whole eye from WT (a) and *Angptl7* KO (b) mice. H&E staining reveals no apparent ocular abnormalities and similar trabecular meshwork and cornea structural organization in WT (c and d) and *Angptl7* KO (e and f) mice. TM: Trabecular Meshwork; C: Cornea; CB: Ciliary body; AC: Anterior chamber. Scale bars represent 100 μm.

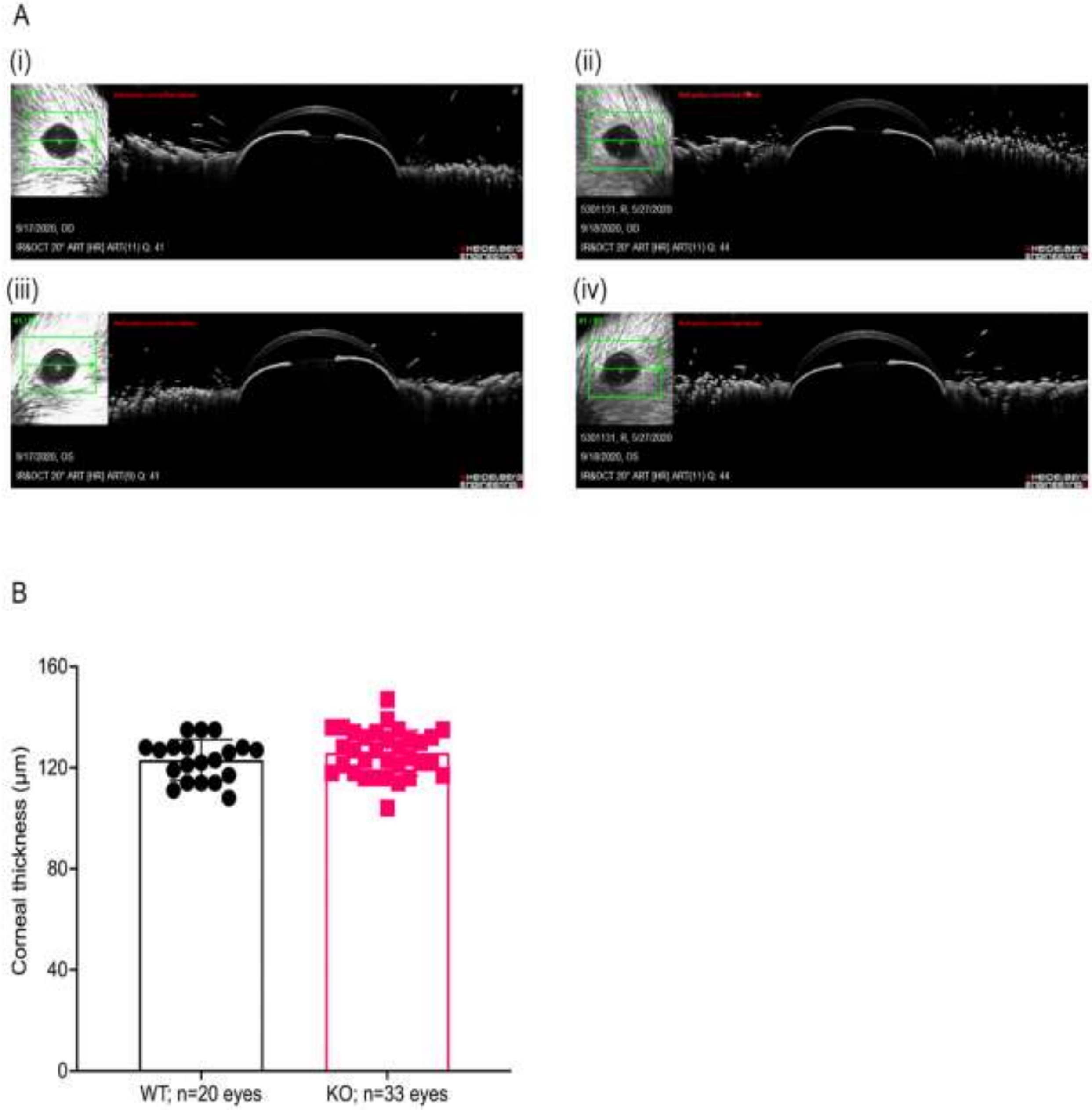

Supplementary Figure 8: Characterization of *Angptl7* KO and WT mouse eyes by OCT. (a) No ocular changes were observed between *Angptl7* KO (ii, iv) and WT (i, iii) mouse eyes on anterior segment OCT. **(b)** *Angptl7* KO and WT mice had similar corneal thickness. Error bars represent the standard deviation.
