# Supplementary material for: ANGPTL7, a therapeutic target for increased intraocular pressure and glaucoma
Source: Commun Biol. 2022 Oct 3;5:1051. doi: 10.1038/s42003-022-03932-6 (PMC9529959; doi:10.1038/s42003-022-03932-6)
Supplement: Supplementary file 3 — Description of Additional Supplementary Files [file 42003_2022_3932_MOESM3_ESM.pdf]

Description of Additional Supplementary Files

File name: Supplementary Data 1
Description: The source data behind all graphs in the paper.

File name: Supplementary Data 2
Description: Uncropped and unedited full blots from Figure 4.
