# Supplementary figures and images for: ANGPTL7, a therapeutic target for increased intraocular pressure and glaucoma
Source: Commun Biol. 2022 Oct 3;5:1051. doi: 10.1038/s42003-022-03932-6 (PMC9529959; doi:10.1038/s42003-022-03932-6)

Supplementary Figure 9: Full blots for main Figure 4.

Whole cell lysate:

ANGPTL7

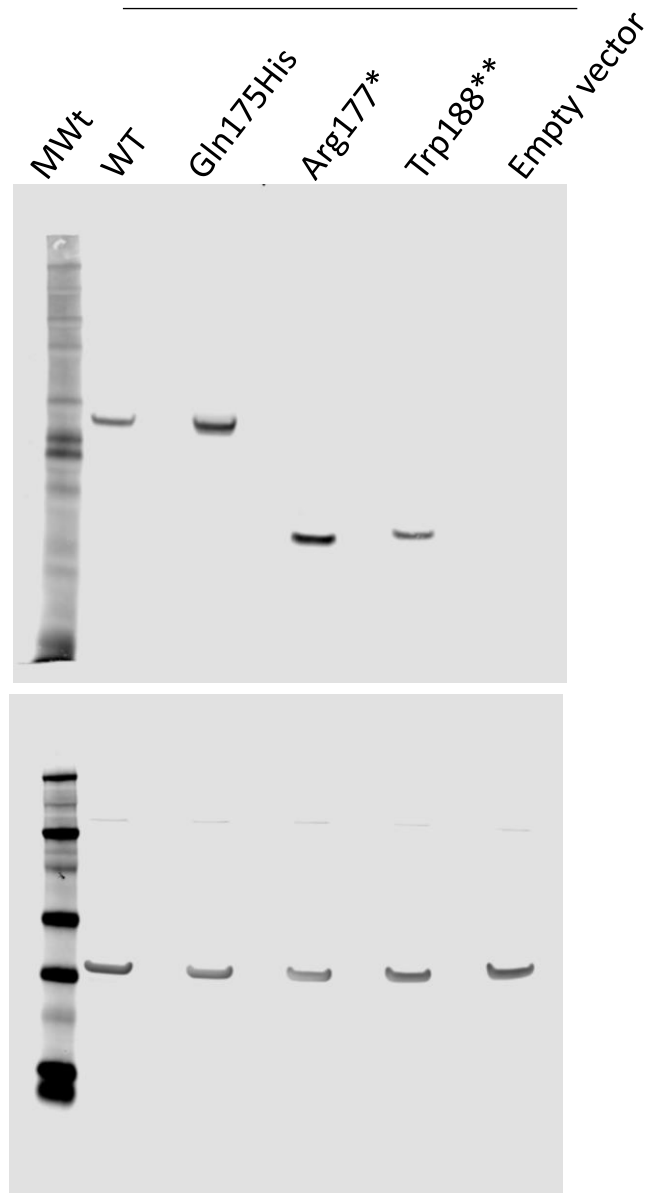

Supernatant:

ANGPTL7

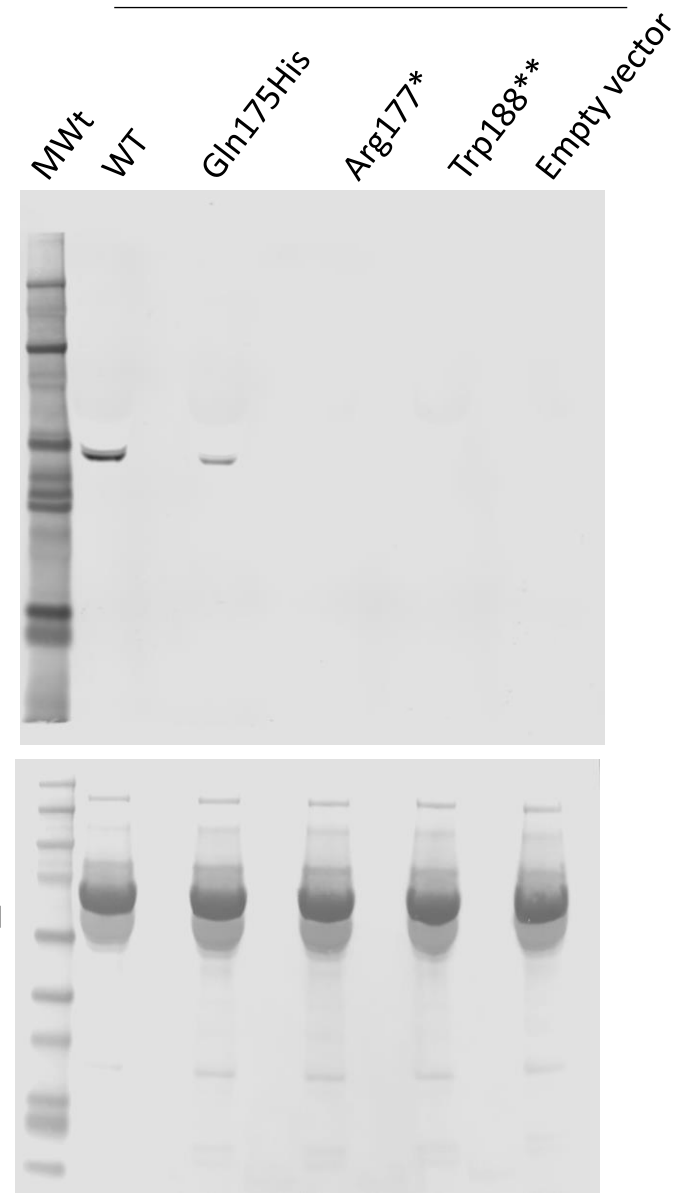

Supplement: Supplementary file 5 — Supplementary Data 2 [file 42003_2022_3932_MOESM5_ESM.pdf]
