# Supplementary material for: ANGPTL7, a therapeutic target for increased intraocular pressure and glaucoma
Source: Commun Biol. 2022 Oct 3;5:1051. doi: 10.1038/s42003-022-03932-6 (PMC9529959; doi:10.1038/s42003-022-03932-6)
Supplement: Supplementary file 6 — Reporting Summary [file 42003_2022_3932_MOESM6_ESM.pdf]

nature portfolio

Corresponding author(s): Giovanni Coppola, Carl Romano, Aris Baras

Last updated by author(s): 2022/08/23

Reporting Summary

Nature Portfolio wishes to improve the reproducibility of the work that we publish. This form provides structure for consistency and transparency in reporting. For further information on Nature Portfolio policies, see our Editorial Policies and the Editorial Policy Checklist.

Statistics

For all statistical analyses, confirm that the following items are present in the figure legend, table legend, main text, or Methods section.

n/a	Confirmed	
☐	☒	The exact sample size (*n*) for each experimental group/condition, given as a discrete number and unit of measurement
☐	☒	A statement on whether measurements were taken from distinct samples or whether the same sample was measured repeatedly
☐	☒	The statistical test(s) used AND whether they are one- or two-sided<br>*Only common tests should be described solely by name; describe more complex techniques in the Methods section.*
☐	☒	A description of all covariates tested
☐	☒	A description of any assumptions or corrections, such as tests of normality and adjustment for multiple comparisons
☐	☒	A full description of the statistical parameters including central tendency (e.g. means) or other basic estimates (e.g. regression coefficient) AND variation (e.g. standard deviation) or associated estimates of uncertainty (e.g. confidence intervals)
☐	☒	For null hypothesis testing, the test statistic (e.g. *F*, *t*, *r*) with confidence intervals, effect sizes, degrees of freedom and *P* value noted<br>*Give P values as exact values whenever suitable.*
☒	☐	For Bayesian analysis, information on the choice of priors and Markov chain Monte Carlo settings
☒	☐	For hierarchical and complex designs, identification of the appropriate level for tests and full reporting of outcomes
☐	☒	Estimates of effect sizes (e.g. Cohen's *d*, Pearson's *r*), indicating how they were calculated

Our web collection on statistics for biologists contains articles on many of the points above.

Software and code

Policy information about availability of computer code

Data collection	For OCT imaging we used the Heidelberg Eye Explorer (Version 1.5.9.0)
Data analysis	Software used for data analysis included Regenie (version 0.0.23), SAIGE, R and Prism.

For manuscripts utilizing custom algorithms or software that are central to the research but not yet described in published literature, software must be made available to editors and reviewers. We strongly encourage code deposition in a community repository (e.g. GitHub). See the Nature Portfolio guidelines for submitting code & software for further information.

Data

Policy information about availability of data

All manuscripts must include a data availability statement. This statement should provide the following information, where applicable:

- Accession codes, unique identifiers, or web links for publicly available datasets
- A description of any restrictions on data availability
- For clinical datasets or third party data, please ensure that the statement adheres to our policy

All whole exome sequencing, genotyping chip, and imputed sequence described in this report are publicly available to registered researchers via the UK Biobank data access protocol. Additional information about registration for access to the data is available at http://www.ukbiobank.ac.uk/register-apply/. Further information about the whole exome sequence is available at http://www.ukbiobank.ac.uk/wp-content/uploads/2019/03/Access_064-UK-Biobank-50k-Exome-Release-FAQ-v3.pdf Detailed information about the chip and imputed sequence is available at: http://www.ukbiobank.ac.uk/wp-content/uploads/2018/03/UKB-Genotyping-and-Imputation-Data-Release-FAQ-v3-2-1.pdf. DiscovEHR and the University of Pennsylvania exome sequencing and genotyping data can be made available to qualified, academic, non-commercial researchers upon request via a Data Transfer Agreement with Geisinger Health System and University of Pennsylvania, respectively. Genetic data for the HUNT, CGPS-CCHS and Estonia cohorts may be made available by contacting the respective institutions. FinnGen R3

data can be accessed at: https://www.finngen.fi/. Regeneron materials described in this manuscript may be available to qualified academic researchers upon request through our portal (https://regeneron.envisionpharma.com/vt_regeneron/). In certain circumstances in which we are unable to provide a particular proprietary reagent, an alternative molecule may be provided that behaves in a similar manner. Additional information about how we share our materials can be obtained by contacting Regeneron's preclinical collaborations email address (preclinical.collaborations@regeneron.com).

Field-specific reporting

Please select the one below that is the best fit for your research. If you are not sure, read the appropriate sections before making your selection.

☒ Life sciences ☐ Behavioural & social sciences ☐ Ecological, evolutionary & environmental sciences

For a reference copy of the document with all sections, see nature.com/documents/nr-reporting-summary-flat.pdf

Life sciences study design

All studies must disclose on these points even when the disclosure is negative.

Sample size	No statistical methods were used to determine sample size. For human genetic analyses, we aimed to maximize the power for detecting associations with rare variants by maximizing the number of glaucoma cases or IOP measurements included in the analysis. For experiments in mice, we used the maximum number of mice that were available, within reason.
Data exclusions	For association analysis of intraocular pressure (IOP), we excluded those individuals who had an IOP greater than 5 standard deviations from the mean of the distributions, or had a difference of ≥10 mmHg in IOP between two eyes or had a glaucoma diagnosis. Individual DNA samples that failed quality control while sequencing, as detailed in methods, were removed from the analysis. No data were excluded in the in vitro and in vivo experiments.
Replication	We performed genetic analyses for the variants of interest across 8 cohorts, as detailed in the results, and observed replication across 3 cohorts for one of the variants. All mouse experiments were performed at least twice and results were consistent between both experiments. In vitro experiments had three biological replicates (all consistent) and data is shown for all three replicates.
Randomization	For genetic analysis, cases and controls assignments were based on self-reported or a clinician diagnosis or on electronic health record information. In knockout mouse characterization, groups were based on genotype (knockout vs. wild-type). In the mouse study involving injection of protein into mouse eyes, inbred mice of the same strain were assigned randomly to vehicle or treatment groups.
Blinding	Mouse experiments were not blinded when measuring IOP and performing injections but were blinded during qPCR and other assays. We relied on repeating the experiments in different cohorts of mice with similar findings. Multiple assays were performed to support the experimental objective and hypothesis.

Reporting for specific materials, systems and methods

We require information from authors about some types of materials, experimental systems and methods used in many studies. Here, indicate whether each material, system or method listed is relevant to your study. If you are not sure if a list item applies to your research, read the appropriate section before selecting a response.

Materials & experimental systems

n/a	Involved in the study	
☐	☒	Antibodies
☐	☒	Eukaryotic cell lines
☒	☐	Palaeontology and archaeology
☐	☒	Animals and other organisms
☐	☒	Human research participants
☒	☐	Clinical data
☒	☐	Dual use research of concern

Methods

n/a	Involved in the study	
☒	☐	ChIP-seq
☒	☐	Flow cytometry
☒	☐	MRI-based neuroimaging

Antibodies

Antibodies used	ANGPTL7 western blot: Cat #10396-1-AP ProteinTech, GAPDH western blot: Cat #G8795 Sigma, ANGPTL7 ELISA: Cat #L3-F50425 Life Sciences
Validation	ProteinTech Cat #10396-1-AP has 3 citations , Sigma Cat #G8795 has 1,126 citations where they have been used.

Eukaryotic cell lines

Policy information about cell lines

Cell line source(s)	HEK293 Hz cell line used was derived at Regeneron Pharmaceuticals Inc.
Authentication	The cell lines used were authenticated.
Mycoplasma contamination	The cell lines tested were negative for mycoplasma contamination.
Commonly misidentified lines (See ICLAC register)	*Name any commonly misidentified cell lines used in the study and provide a rationale for their use.*

Animals and other organisms

Policy information about studies involving animals; ARRIVE guidelines recommended for reporting animal research

Laboratory animals	C57BL6 and C57BL/6NTac adult mice (8-10 weeks), males and females, were used for in vivo experiments.
Wild animals	The study did not involve wild animals.
Field-collected samples	The study did not involve samples collected from the field.
Ethics oversight	All animal protocols were approved by the Institutional Animal Care and Use Committee in accordance with the Regeneron's Institutional Animal Care and Use Committee (IACUC) and the Association for Research in Vision and Ophthalmology (ARVO) Statement for the Use of Animals in Ophthalmic and Vision Research.

Note that full information on the approval of the study protocol must also be provided in the manuscript.

Human research participants

Policy information about studies involving human research participants

Population characteristics	Participants were of European or African ancestry, median age was over 55 years and were sightly enriched in females. Age and sex were included as covariates in all genetic association analyses.
Recruitment	Participants were recruited through health systems, biobanks and projects specifically designed to study glaucoma. There can be some bias in the cohort based on ascertainment as individuals identified in hospital settings or by electronic health records may have more severe disease as opposed to individuals in a healthy population setting self-reporting the disease. However, since glaucoma requires the diagnosis by an ophthalmologist, we do not anticipate that self-reporting the disease in a cohort would have a major impact on the results. Participants in the IOP analyses were recruited through a biobank, a hospital system and through projects specifically designed to study glaucoma. IOP measurements can be influenced by use of glaucoma medications. To control for this, we performed genetic analysis only on individuals who did not have a glaucoma diagnosis or were not on glaucoma medications at the time of IOP measurement.
Ethics oversight	All participants provided informed consent, and studies were approved by the individual IRBs at the respective institutions. UK Biobank has approval from the North West Multi-centre Research Ethics Committee (MREC), which covers the UK. It also sought the approval in England and Wales from the Patient Information Advisory Group (PIAG) for gaining access to information that would allow it to invite people to participate. The DiscovEHR study was approved by the Institutional Review Board (IRB) at Geisinger. The BioMe Biobank is an ongoing research biorepository approved by the Icahn School of Medicine at Mount Sinai's IRB. The Ethical Committee at Lund University approved the Malmo Diet and Cancer Study (LU 51-90) and all the participants provided a written informed consent. The CGPS study (H-KF-01-144/01) have obtained approval from the Ethics Committee of the Capital Region and from the Danish Data Protection Agency. Research at Estonian Biobank is regulated by Human Gene Research Act and all participants have signed a broad informed consent. IRB approval for current study was granted by Research Ethics Committee of University of Tartu, approval nr 236/T-23. For the POAAGG study, Approval to enroll and to recontact subjects was obtained from the University of Pennsylvania IRB. The Finngen Biobank was evaluated and approved by the Coordinating Ethics Committee of the Helsinki and Uusimaa Hospital District.

Note that full information on the approval of the study protocol must also be provided in the manuscript.
